# Direct versus Indirect Treatment for Preschool Children who Stutter: The RESTART Randomized Trial

**DOI:** 10.1371/journal.pone.0133758

**Published:** 2015-07-28

**Authors:** Caroline de Sonneville-Koedoot, Elly Stolk, Toni Rietveld, Marie-Christine Franken

**Affiliations:** 1 Institute of Health Policy and Management, Erasmus University Rotterdam, Rotterdam, the Netherlands; 2 Speech and Hearing Center, Department of Otorhinolaryngology, Sophia Children’s Hospital, Erasmus University Medical Center, Rotterdam, the Netherlands; 3 Department of Linguistics, Radboud University Nijmegen, Nijmegen, the Netherlands; The National Institute for Health Innovation, NEW ZEALAND

## Abstract

**Objective:**

Stuttering is a common childhood disorder. There is limited high quality evidence regarding options for best treatment. The aim of the study was to compare the effectiveness of direct treatment with indirect treatment in preschool children who stutter.

**Methods:**

In this multicenter randomized controlled trial with an 18 month follow-up, preschool children who stutter who were referred for treatment were randomized to *direct* treatment (Lidcombe Program; n = 99) or *indirect* treatment (RESTART-DCM treatment; n = 100). Main inclusion criteria were age 3–6 years, ≥3% syllables stuttered (%SS), and time since onset ≥6 months. The primary outcome was the percentage of non-stuttering children at 18 months. Secondary outcomes included stuttering frequency (%SS), stuttering severity ratings by the parents and therapist, severity rating by the child, health-related quality of life, emotional and behavioral problems, and speech attitude.

**Results:**

Percentage of non-stuttering children for direct treatment was 76.5% (65/85) versus 71.4% (65/91) for indirect treatment (Odds Ratio (OR), 0.6; 95% CI, 0.1–2.4, *p* = .42). At 3 months, children treated by direct treatment showed a greater decline in %SS (significant interaction time x therapy: β = -1.89; t(282.82) = -2.807, *p* = .005). At 18 months, stuttering frequency was 1.2% (SD 2.1) for direct treatment and 1.5% (SD 2.1) for indirect treatment. Direct treatment had slightly better scores on most other secondary outcome measures, but no differences between treatment approaches were significant.

**Conclusions:**

Direct treatment decreased stuttering more quickly during the first three months of treatment. At 18 months, however, clinical outcomes for direct and indirect treatment were comparable. These results imply that at 18 months post treatment onset, both treatments are roughly equal in treating developmental stuttering in ways that surpass expectations of natural recovery. Follow-up data are needed to confirm these findings in the longer term.

**Trial Registration:**

isrctn.org ISRCTN24362190

## Introduction

Developmental stuttering is a prevalent childhood disorder. The incidence rate is 5 to 11% in preschool years [1,2]. The cause of stuttering is unknown, although recent research indicates that structural and functional brain anomalies underlie the disorder [3–6], with a strong genetic involvement [7–10]. Many children recover spontaneously; about 63% at 3 years post onset [1,11]. Knowledge of factors that favor the chance for recovery [11,12] can help pediatricians and speech-language pathologists (SLPs) to identify children at risk for chronic stuttering [13]. Nevertheless, the chance for recovery cannot be predicted for an individual child. Since chances for full recovery diminish when stuttering has been present for 15 months [14] and persistent stuttering in adolescents and adults can have a serious mental and social impact [15–17], treatment is generally recommended to start before the age of 6 years [2,18]. However, the evidence base for the effectiveness of current therapies for preschool children who stutter is surprisingly weak as well as unbalanced in terms of published reports [19].

For about three decades, many preschool children who stutter around the world have been treated according to an *indirect*, multifactorial treatment approach, like treatment based on the Demands and Capacities Model (DCM) [20,21]. This approach aims to decrease demands set by the environment (e.g., parents are trained to slow down their habitual speech rate) and the child him- or herself (e.g., desensitization for disfluency), and increase the child’s capacities for speaking fluently (e.g., accurate and smooth speech motor movements that are age-appropriate) to arrive at a favorable balance between demands and capacities, eventually resulting in fluent speech. Since 2000, an increasing number of children have been treated according to a *direct* operant treatment approach: the Lidcombe Program (LP) for early intervention [22,23]. This direct approach teaches parents to give verbal contingencies after fluent and stuttered speech. With the limited data available at present, the direct LP offers the best evidence-based intervention for preschool children who stutter [19]. However, the long-term effectiveness of this treatment is still unclear [24]. More importantly, comparative effectiveness to current standard treatment has not yet been established; yet child health policy-makers, pediatricians and SLPs need this information to decide upon reimbursement and treatment choice. This is for instance illustrated by a recent proposal of the national speech-language pathology association of Australia (Speech Pathology Australia) to only fund treatment by the LP [25]. Therefore, the aim of the current study was to compare the effectiveness of direct versus indirect stuttering treatment in preschool children during an 18 month follow-up.

## Methods

### Study design, participants and setting

This parallel group randomized trial named RESTART (the Rotterdam Evaluation Study of Stuttering Therapy in preschool children- a Randomized Trial) included 199 preschool children who stutter, who were registered at one of the 20 participating speech clinics (including 24 SLPs) throughout the Netherlands. Eligible participants were children (1) aged 3.0–6.3 years, (2) with a stuttering severity rating ≥ 2 (‘mild’) on an 8-point scale [11] provided by the parent (3) and by the clinician, (4) who stuttered ≥ 3% of syllables and (5) for at least 6 months. The inclusion criterion of at least 3% syllables stuttered (SS) had replaced the original criterion of ‘at least 3.3% Stuttering Like Disfluencies (SLD)’ shortly before the start of the trial. This was based on critics on the SLD measure in literature and on the results of a study into the validity of the SLD measure that we conducted at our center. Exclusion criteria were a diagnosis of an emotional, behavioral, learning or neurological disorder, or a lack of proficiency in Dutch for children or parents. The exclusion criterion of having received treatment for stuttering during the past year was omitted after 5 months, since it was noticed that by excluding these children, the external validity would be restricted. All SLPs were trained and experienced in both treatments. DCM based treatment training is included in the regular clinical education in the Netherlands, and all but one SLP had additionally been trained in the assessment and treatment of children who stutter to become a certified fluency expert recognized by the Dutch association of stuttering therapy (NVST). To ensure a uniform application of DCM based treatment, a treatment manual was developed in collaboration with all participating clinicians prior to the start of the trial. In addition, all SLPs had gone through a three day LP course taught by a LP Consortium trainer and had been certified to provide LP therapy. They had on average 15 years of experience with DCM based treatment (range 7–21 years) and 3.7 years with the LP (range 1.5–7.6 years). Therapists’ fidelity to treatment was monitored in 3-monthly intervision meetings, regular telephone contacts with the research team, and by registration forms on the content and amount of treatment filled in by the SLPs and checked by the research team. The intervision meetings were chaired by a LP consortium trainer and a DCM trainer. The trial was approved by the Ethics Committee of the Erasmus MC and registered at isrctn.org (ISRCTN24362190). Written informed consent was obtained from all parents. The trial protocol and supporting CONSORT checklist are available as supporting information: see S1 CONSORT Checklist and S1 Protocol.

### Interventions

#### Direct treatment: The Lidcombe Program

The Lidcombe Program (LP) is a behavioral treatment based on the premise that stuttering is an operant behavior that can be targeted by contingencies. The LP is administered by parents under the direction of a clinician. Children allocated to the LP were treated according to the LP manual [22]. Parents were trained to deliver verbal contingencies in conversations with their child (e.g., “That was smooth” or “Were there any bumpy words?”) in a 5:1 ratio for stutter-free and stuttered speech. During the first stage of the program, the parent delivered contingencies during structured conversations of 10–15 minutes once or twice a day. The speech clinic was attended once a week. This continued until stuttering either disappeared or reached an extremely low level (≤1% of syllables stuttered). During the second stage, the use of verbal contingencies as well as the number of clinic visits was gradually reduced, provided that fluency was maintained.

#### Indirect treatment: The RESTART Demands and Capacities Model based treatment

RESTART Demands and Capacities Model based treatment (RESTART-DCM) is premised on the idea that positive changes in the child’s functioning and/or in the environment will lead to a reduction of stuttering. Following the RESTART-DCM manual [26], parents were trained to decrease relevant motoric, linguistic, emotional or cognitive demands, thereby reduce communicative pressure on the child (e.g., parents slowing down their habitual speech rate). If deemed necessary, the child’s capacities for fluency were subsequently trained (e.g., improving the child’s speech motor movements or word-finding capacity). Parents were required to give their child their undivided attention and practice home assignments 15 minutes a day, for a minimum of 5 days a week. Treatment was gradually reduced if the child showed acceptable speech, parents had mastered implementing a fluency enhancing environment and knew what to do if a relapse occurred.

### Randomization and blinding

A minimization software program (MINIM2) [27] was used by the principal investigator (CdeS) to allocate children to one of the treatment arms, according to factors known or thought of to be related to treatment outcome [28]: gender, stuttering severity in the clinic (based on the SSI-3 score) [29], time since onset (TSO; 6–12, 13–18, 19+ months), a first, second, or third degree relative with persistent stuttering (yes, no) and/or a history of recovered stuttering (yes, no), stuttering treatment during the past 12 months (yes, no), and SLP. Three stuttering severity categories were distinguished: (1) mild (SSI-3 score: 10–16); (2) moderate (SSI-3 score: 17–26); severe (SSI-3 score: 27+). For each participant, treatment allocation depended on the characteristics of the children already enrolled [28]. Judges of stuttering frequency were blinded to treatment allocation and measurement moment.

### Outcome assessment

The primary outcome measure was the percentage of non-stuttering children at 18 months, operationalized as ≤1.5% syllables stuttered (SS). This criterion was obtained by applying a conversion ratio of 1.15 to the mean percentage of stuttered word disfluencies in children who do not stutter [30,31]. Parents were requested to make three audio recordings of 10–15 minutes each in a period of two weeks: one sample of their child speaking to a parent at home, one to a non-family member at home and one to a non-family member away from home [32–34].

Secondary outcome measures assessed at baseline, and at 3, 6, 12 and 18 months after start of treatment, were the frequency of stuttering (%SS), a severity rating of stuttering by the parent on an 8-point scale[11], and parents´ valuation of their child´s health-related quality of life on a proxy version of the EuroQoL EQ-VAS [35] with anchor points 0 (worst imaginable health) and 100 (best imaginable health). Secondary outcome measures assessed at baseline and 18 months were the speech attitude of the child (KiddyCAT) [36] and emotional and behavioral problems measured by the Child Behavior Checklist (CBCL) [37]. The latter consists of the scales Internalizing (anxiety, depression, withdrawal, and somatic complaints), Externalizing (aggressive and delinquent behavior), and Total problem behavior [37]. At 18 months both the SLP and the child provided a stuttering severity rating: the SLP on an 8-point scale [11], the child on a 4-point scale where 1 = I do not stutter anymore and 4 = I stutter a lot.

Eight SLPs not involved in the study were trained to determine the %SS of the samples in real time with sufficient intrajudge reliability, using an electronic, button press counter. To ensure sufficient interjudge reliability, 64% of all samples were scored by at least two raters. Disagreements in ratings were discussed and a third, blinded senior rater was consulted in rare cases where no agreement could be reached (cf. Boberg & Kully [38]).

### Statistical analysis

An a priori power calculation to detect a difference of 15% in percentage of non-stuttering children (80 versus 95%) with a power of 80% in a 2-tailed test at a significance level of .05 and allowing a 22% drop-out rate, resulted in a sample size of 98 in each group. Baseline factors were characterized as medians, means and standard deviations for continuous variables and as frequency distributions for categorical variables. Baseline comparisons between treatment groups and between survivors and drop-outs were assessed using χ^2^ tests and independent t-tests. Participants were analyzed in the group to which they were randomized.

The effect of treatment on the primary outcome measure was analyzed by χ^2^ tests and logistic regression analysis (ENTER method). The regression analysis included the main effect of therapy and the interaction terms therapy*age in years, therapy*stuttering severity (SSI-3 score), and therapy*TSO. Confidence intervals around the obtained percentages of children classified as non-stuttering were calculated according to the method of Wilson [39,40], using a website calculator (http://www.vassarstats.net/prop1.html). In a sensitivity analysis, cut-off scores of 1% SS and 2% SS were applied to further assess the robustness of the primary outcome.

For the secondary outcomes assessed at all measurement moments (%SS, parental rating of stuttering severity, and EQ-VAS) and at baseline and 18 months (KiddyCAT and CBCL), we applied a longitudinal repeated-measures mixed effects model with random intercepts, assuming missing at random. Participant was included as a random predictor; fixed predictors were therapy, and 4 cross-products as interaction terms: time*therapy, and time*therapy*age, severity, and TSO, respectively. An unstructured covariance matrix was assumed for the error as a more plausible autoregressive covariance structure did not provide a better fit. This approach was also used at level 2 of the model. Since the data on %SS did not meet the assumptions needed to calculate CIs for the intraclass correlation coefficient (ICC), interjudge reliability of the speech samples was assessed using Krippendorff's alpha [41] with the option 'interval data' for the macro developed by Hayes (2013) [42]. For the outcome %SS, an additional analysis was conducted into the progression in the first 3 months. CBCL outcomes at 18 months were analyzed separately using ANOVA-analysis. Secondary outcome measures only assessed at 18 months (severity ratings by clinician and child) were compared by independent t-tests. For all secondary outcomes, unadjusted and Holm-adjusted [43] *p*-values are presented, using an overall level of significance of α = .05 (2-sided). The Holm’s correction is generally considered a good alternative to the conservative Bonferroni approach [44]. Each p_j_ is compared to α/(n-j+1); that is: the smallest p_j_ (j = 1) is compared to α/n, the next smallest to α/(n-1) etc.

Treatment intensity was compared by independent t-test, and a χ^2^ test was conducted to compare the number of children on treatment at the endpoint of the trial. For analysis of the questionnaires, instructions offered in the manuals were followed. All analyses were carried out in SPSS 20 (Armonk, NY: IBM Corp.).

## Results

### Participants

**Fig 1:**
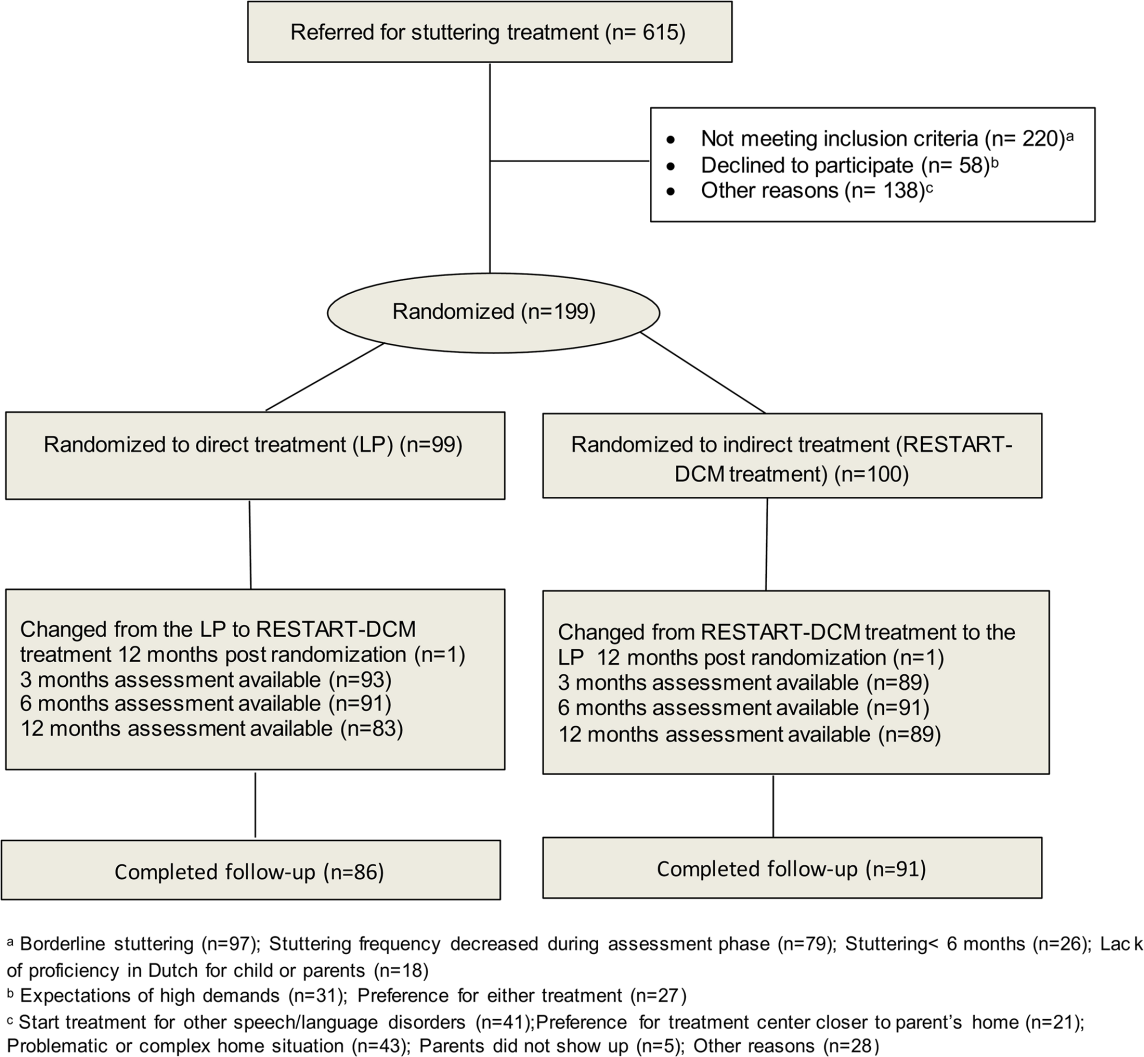
RESTART Trial Flow Diagram.

Children were enrolled between September 2007 and June 2010. Of 615 children referred for treatment, 416 were not eligible for various reasons (Fig 1). In total 199 children met the inclusion criteria. One child was found ineligible after inclusion and therefore excluded from all analyses (Fig 1). Baseline characteristics did not differ between treatment groups (Table 1). In the LP group 12 children were lost to follow-up as compared to 9 children in the RESTART-DCM group (n = 21, 11% drop out rate). Children who were lost to follow-up did not significantly differ on any baseline characteristics (age, gender, ethnicity, educational level of parent, SSI-3 score, %SS, TSO, parental ratings, stuttering in family, prior treatment for stuttering) from children who completed the trial (*p*-values ranging from .11 to .91). For 191 children, at least one outcome measurement after the start of treatment was available.

**Table 1:** Baseline Characteristics of Participants by Treatment Group.

Characteristic	Lidcombe Program (n = 98)^a^	RESTART-DCM (n = 100)^a^
Age in months, median; mean (SD)	51.0; 51.5 (9.5)	52.0; 54.1 (11.1)
Age in years		
3^b^	41 (41.8)	37 (37.0)
4	39 (39.8)	31 (31.0)
5–6	18 (18.4)	32 (32.0)
Male	68 (69.4)	70 (70.0)
SSI-3 score		
mild^c^	32 (32.7)	31 (31.0)
moderate	47 (48.0)	51 (51.0)
severe	19 (19.4)	18 (18.0)
% SS, median; mean (SD)^d^	4.9; 6.2 (4.4)	4.0; 5.3 (4.3)
Time since onset		
6–12 months	43 (43.9)	45 (45.0)
13–18 months	25 (25.5)	22 (22.0)
19+ months	30 (30.6)	33 (33.0)
Family history of persistency^e^	45 (45.9)	45 (45.0)
Family history of recovery^e^	27 (27.6)	25 (25.0)
Prior treatment for stuttering	8 (8.2)	6 (6.0)

^a^ Data are shown as No. (%) unless specified otherwise.

^b^ One child in the LP group was 2.11 years at time of inclusion.

^c^ Children with a stuttering frequency < 3% in the therapy setting but ≥ 3% in the home setting were included in the group ‘mild stuttering’ (n = 26).

^d^ For one child in the RESTART-DCM group %SS on baseline was not available.

^e^ For one child in the LP group information on family history of stuttering was not available.

### Speech samples

The mean number of available audio samples for a child at a measurement moment was 2.9 (range 1–6). At least 85% of all samples had a length of ≥300 syllables. The mean intrajudge reliability [45] of measurement of %SS was 83%. Krippendorff’s alpha for samples with 2 ratings at baseline and after 3, 6, 12 and 18 months, respectively, was 0.849, 0.896, 0.817, 0.795, and 0.830; all significant, with significance obtained by bootstrapping. All scores represent good reliability [46].

### Primary outcome

At 18 months, audiotapes were available for 173 children. For 1 child in the LP and 2 in the RESTART-DCM group audiotapes were missing and replaced by videotapes made in the clinic. For 1 child in the LP group, both audio and videotapes were lacking. Thus, the final analysis at 18 months was based on 176 children. In total, 76.5% (65/85; 95%CI: 66.4–84.2) of children in the LP group were classified as non-stuttering at 18 months compared to 71.4% (65/91; 95%CI: 61.4–79.7) of children in the RESTART-DCM group. This difference was statistically non-significant (χ^2^(1) = 0.579, *p* = .45). Nor did logistic regression analysis indicate therapy or other factors as significant predictors of being classified as non-stuttering (therapy: OR, 0.6; 95% CI, 0.1–2.4; *p* = .42; Table 2). Applying cut-off criteria of 1% SS and 2% SS did not significantly affect the results.

**Table 2:** Primary and Secondary Outcomes at Baseline and 18-month Follow Up.

		Baseline			18 months			Parameter	Estimate (95% CI)	z	Unadjusted *p*-value	Adjusted *p*-value
	Outcome measure	Number of participants	LP	RESTART-DCM	Number of participants	LP	RESTART-DCM					
% recovery		197	-	-	176	76.5	71.4					
	Therapy type							OR	0.6 (0.1; 2.4)	-	.42	-
	Therapy type x Age(1)^a^							OR	2.8 (0.7; 11.5)	-	.16	-
	Therapy type x Age(2)^a^							OR	1.2 (0.4; 3.8)	-	.75	-
	Therapy type x Severity(1)^b^							OR	0.6 (0.1; 2.7)	-	.50	-
	Therapy type x Severity(2)^b^							OR	0.8 (0.2; 3.0)	-	.71	-
	Therapy type x TSO(1)^c^							OR	1.0 (0.3; 3.4)	-	.98	-
	Therapy type x TSO(2)^c^							OR	4.6 (0.8; 27.2)	-	.09	-
% SS		197	6.2 (4.4)	5.3 (4.3)	176	1.2 (2.1)	1.5 (2.1)					
	Therapy type							β	0.62 (-0.65; 1.89)	0.96	.34	-
	Time							β	-0.76 (-1.21; -0.31)	-3.30	.001	.002
	Time x Therapy type							β	-0.51 (-0.86; -0.16)	-2.90	.004	.008
	Time x Therapy type x Age							β	0.04 (-0.02; 0.10)	1.40	.16	.32
	Time x Therapy type x Severity							β	0.04 (-0.01; 0.10)	1.57	.12	.13
	Time x Therapy type x TSO							β	0.05 (0.002; 0.11)	2.04	.04	0.13
Parental severity rating		189	4.4 (1.0)	4.3 (1.0)	176	1.0 (1.4)	1.4 (1.5)					
	Therapy type							β	0.13 (-0.25; 0.51)	0.68	.50	-
	Time							β	-0.67 (-0.85; -0.50)	-7.61	< .001	< .001
	Time x Therapy type							β	-0.38 (-0.55; -0.22)	-4.62	< .001	< .001
	Time x Therapy type x Age							β	0.07 (0.03; 0.10)	3.66	< .001	< .001
	Time x Therapy type x Severity							β	0.04 (0.00; 0.07)	2.15	.03	.10
	Time x Therapy type x TSO							β	0.00 (-0.02; 0.04)	0.51	.61	1
EQ-VAS		182	88.0 (10.2)	88.4 (10.1)	168	91.5 (9.7)	90.5 (10.2)					
	Therapy type							β	-0.09 (-3.22; 3.04)	-0.06	.96	-
	Time							β	0.18 (-0.99; 1.37)	0.31	.76	.76
	Time x Therapy type							β	0.35 (-0.79; 1.49)	0.60	.55	.55
	Time x Therapy type x Age							β	0.04 (-0.15; 0.35)	0.78	.44	.44
	Time x Therapy type x Severity							β	0.10 (-0.47; 0.01)	-1.86	.06	.13
	Time x Therapy type x TSO							β	-0.23 (-0.18; 0.27)	0.37	.71	1
CBCL Internal score		193	10.4 (7.9)	7.4 (5.9)	173	5.5 (5.2)	4.2 (4.5)					
	Therapy type							β	4.80 (1.21; 8.39)	2.63	.009	.02
	Time							β	-1.17 (-4.16; 1.82)	-0.77	.44	-
	Time x Therapy type							β	-0.77 (-3.00; 1.46)	-0.68	.50	-
	Time x Therapy type x Age							β	-0.29 (-0.63; 0.05)	-0.82	.10	.29
	Time x Therapy type x Severity							β	0.14 (-0.19; 0.46)	-1.66	.41	-
	Time x Therapy type x TSO							β	-0.13 (-0.44; 0.18)	-0.83	.41	-
CBCL External score		193	13.6 (7.4)	11.2 (7.6)	173	7.1 (5.8)	6.2 (5.7)					
	Therapy type							β	3.93 (0.37; 7.49)	2.18	.03	.03
	Time							β	-2.85 (-5.68; -0.02)	-1.99	.05	-
	Time x Therapy type							β	0.97 (-1.25; 3.19)	0.86	.39	-
	Time x Therapy type x Age							β	-0.68 (-1.06; -0.30)	-3.51	.001	.004
	Time x Therapy type x Severity							β	-0.03 (-0.40; 0.34)	-0.17	.86	-
	Time x Therapy type x TSO							β	-0.07 (-0.41; 0.28)	-0.39	.70	-
CBCL Total problem score		193	36.2 (20.6)	27.9 (17.6)	173	21.8 (15.4)	18.6 (13.8)					
	Therapy type							β	13.40 (3.75; 23.03)	2.74	.007	.02
	Time							β	-3.52 (-11.63; 4.59)	-0.86	.39	-
	Time x Therapy type							β	-3.73 (-9.97; 2.50)	-1.18	.24	-
	Time x Therapy type x Age							β	-0.50 (-1.31; 0.75)	-0.54	.59	.59
	Time x Therapy type x Severity							β	-0.28 (-0.73; 1.25)	0.52	.60	-
	Time x Therapy type x TSO							β	0.26 (-1.43; 0.43)	-1.06	.29	-
KiddyCAT^d^		182	3.6 (2.5)	3.9 (2.9)	116	1.2 (1.5)	2.0 (2.1)					
	Therapy type							β	0.15 (-1.32; 1.63)	0.20	.84	-
	Time							β	-1.35 (-2.77; 0.07)	-1.87	.06	-
	Time x Therapy type							β	-0.88 (-1.93; 0.17)	-1.65	.10	.40
	Time x Therapy type x Age							β	0.09 (-0.07; 0.25)	1.13	.26	.52
	Time x Therapy type x Severity							β	0.04 (-0.11; 0.18)	0.49	.62	-
Severity rating by clinician		NA	NA	NA	168	1.1 (1.4)	1.4 (1.4)					
	Therapy type							β	0.00 (0.00; 0.00)	-	.93	-
	Therapy type x Age							β	0.08 (0.01; 0.13)	-	.01	.01
	Therapy type x Severity							β	0.04 (0.00; 0.09)	-	.14	-
	Therapy type x TSO							β	0.02 (0.00; 0.05)	-	.50	-
Severity rating by child		NA	NA	NA	168	1.4 (0.5)	1.4 (0.5)					
	Therapy type							β	0.00 (0.00; 0.03)	-	.49	-
	Therapy type x Age							β	0.09 (0.01; 0.14)	-	.006	.01
	Therapy type x Severity							β	0.04 (0.00; 0.09)	-	.14	-
	Therapy type x TSO							β	0.01 (0.00; 0.01)	-	.88	-

^a^ Age(1) refers to age 4 years; age(2) refers to age 5–6 years.

^b^ SSI(1) refers to moderate stuttering severity; SSI(2) refers to severe stuttering severity.

^c^ TSO(1) refers to TSO 13–18 months; TSO(2) refers to TSO 19+ months.

^d^ The KiddyCAT was only applicable for preschool children. Therefore, the effect of TSO could not be precisely estimated and TSO was left out in the analysis.

### Secondary outcomes

The results for all secondary outcome measures at baseline and 18 months and the results for the mixed model analyses are presented in Table 2. For the outcome %SS, the effect of therapy type was non-significant. However, a significant interaction between time and therapy type was detected (adjusted *p* = .008), indicating that the %SS differed for therapy groups at different time points. The effect of time was also significant (adjusted *p* = .002), indicating that in both treatment groups the average %SS decreased significantly over time. Effect sizes were small (Table 2).

**Fig 2:**
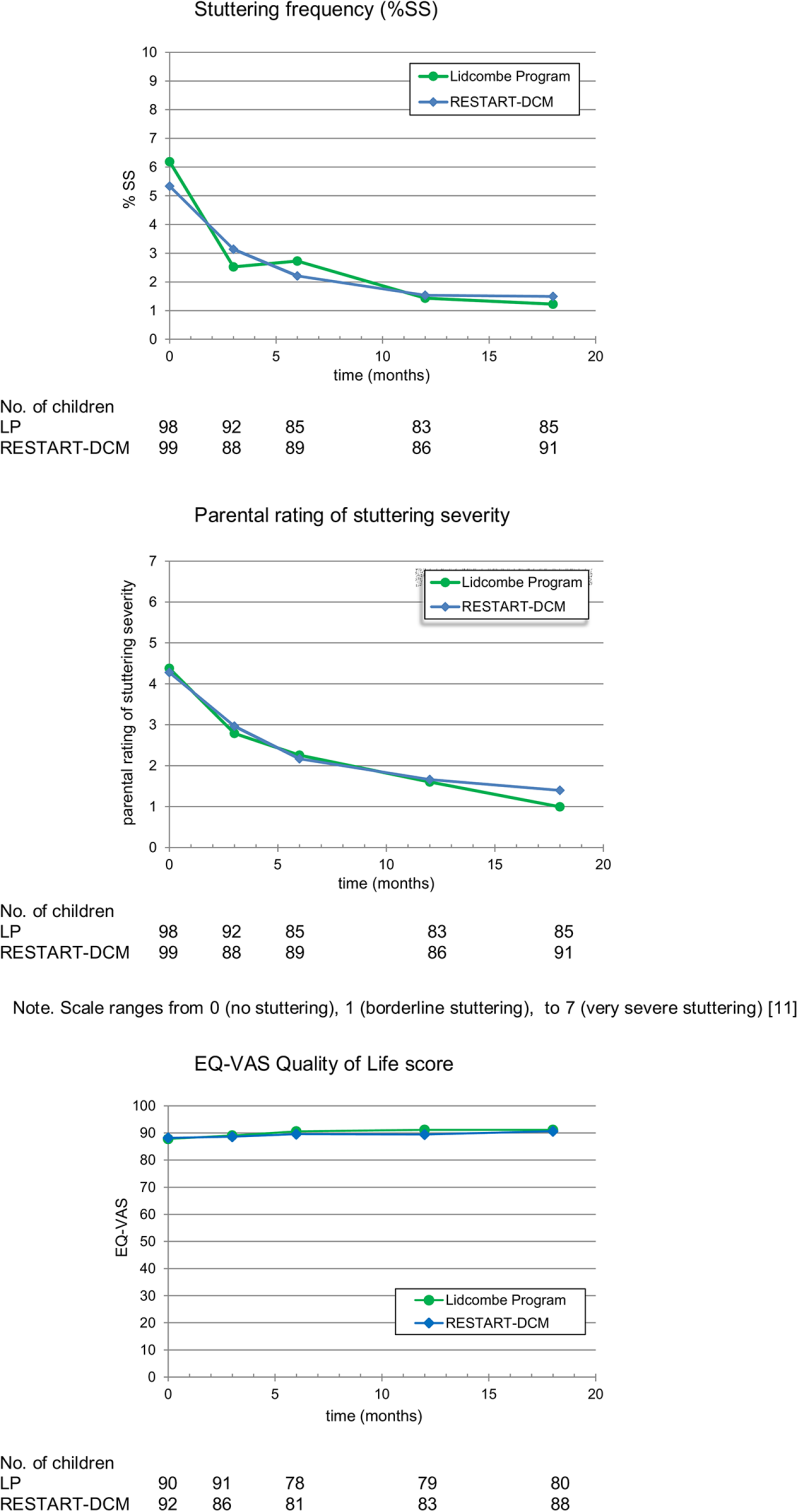
Change in Three Secondary Outcome Measures During 18-month Follow Up.

**Fig 3:**
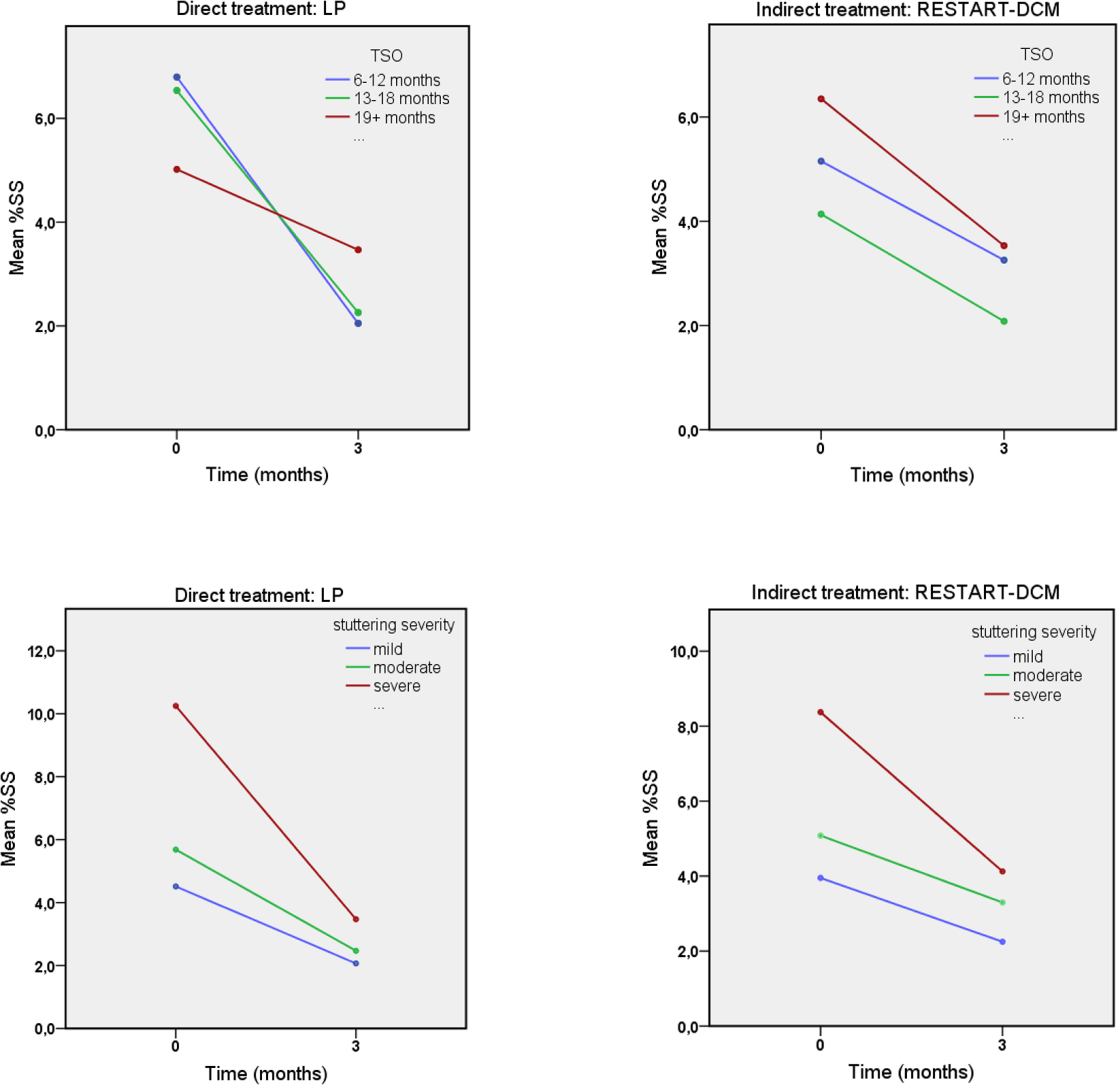
Change in %SS During first 3 Months.

Fig 2 shows that in both groups most improvement in %SS occurred in the first 3 months of therapy. For this interval, an effect of therapy type was found (β = 2.30; t(217.38) = 2.10, *p* = .04), as well as a significant interaction between time and therapy type (β = -1.89; t(282.82) = -2.81, *p* = .005). Compared to the RESTART-DCM group, the LP group had a slightly higher mean %SS at baseline and showed a greater decline, resulting in a lower %SS at 3 months. Significant interactions with very small effect sizes were also present between time, therapy type, and stuttering severity (β = 0.25; t(173.94) = 2.51, adjusted *p* = .01) and time, therapy type, and TSO (β = -0.21; t(172.85) = 2.40, adjusted *p* = .02) (Fig 3).

For the outcome parental rating of stuttering severity, a significant effect of time (adjusted *p*< .001) as well as a significant interaction between time and therapy type (adjusted *p*< .001) was detected. Fig 2 shows a slightly greater decline in scores for the LP group over the period of 18 months. The interaction between time, therapy type and age was significant (adjusted *p*< .001) but showed a very small effect size (Table 2). For the outcomes EQ-VAS and KiddyCAT, no significant effect of therapy type or any other factor was found (Table 2; Fig 2).

For all CBCL scale scores, the factor therapy type was significant (Table 2), but this effect was attributable to significantly higher scores for the LP group at baseline. At 18 months, no significant differences were found (Internal scale: F_(1,196)_ = -1.04, unadjusted *p* = .32, partial eta squared = .006; External scale: F_(1,196)_ = 1.04, unadjusted *p* = .31, partial eta squared = .006; Total problem scale: F_(1,196)_ = 1.12, unadjusted *p* = .29, partial eta squared = .006). For the CBCL External scale, a significant interaction with a small effect size was established between time, therapy type and age: older children showed a greater decline in score, particularly in the LP group.

For the severity rating by the clinician as well as by the child at 18 months, significant interactions between therapy type and age were established (Clinician: adjusted *p* = .01; Child: adjusted *p* = .01). However the small eta-squared values (0.079 and 0.088, respectively) suggest that these differences are negligible.

### Treatment intensity

The number of treatment sessions and treatment hours did not differ significantly between groups (Table 3). At 18 months, 27.6% (27/99) children in the LP group compared to 35.0% (35/100) children in the RESTART-DCM group were still on treatment, a difference that was also not statistically significant (*χ*2(1) = 1.277, *p* = .26).

**Table 3:** Treatment Intensity by Treatment Group.

	**LP (N = 98)**	**RESTART-DCM (N = 97)**	***p*-value**
Number of treatment sessions, median; mean (SD; SE) [range]	21; 22.2 (11.2; 1.1) [2–51]	17; 19.5 (10.3; 1.0) [2–59]	.08
	**LP (N = 95)**	**RESTART-DCM (N = 93)**	
Number of treatment hours, median; mean (SD; SE) [range]	18.3; 19.6 (10.9; 1.1) [1.4–51]	15.5; 18.0 (9.7; 1.0) [3.0–55.2]	.20

## Discussion

The RESTART-trial found that both direct and indirect treatment for preschool children who stutter reduced stuttering during 18 months of follow-up. The direct approach reduced stuttering frequency more quickly during the first three months of treatment, however, the difference was not significant anymore by 18 months. Most outcome measures were slightly in favor of the direct approach (LP), but the few significant interaction terms were deemed negligible due to their small effect sizes. For most children, stuttering frequency plateaued after three months, while about 30% of children were still on treatment after 18 months.

The direct LP and indirect RESTART-DCM treatment are based on different premises and assumptions regarding mechanisms underlying treatment effect (i.e., delivering verbal contingencies versus balancing demands and capacities for fluent speech, respectively). However, since results for both treatments were comparable, it could be hypothesized that their common components have a larger influence on recovery than their unique components (cf. Imel & Wampold [47]). In psychotherapy and counseling, this is known as the “dodo bird phenomenon” [48]. According to this hypothesis, treatments that are intended to be therapeutic are equally efficacious. Studies suggest that 30–70% of therapy outcome can be attributed to common factors, including good therapeutic relationships [47]. Unfortunately, little is known about the unique mechanisms that lead to change in stuttering behavior in both treatments [49–51]. Common components of the LP and RESTART-DCM treatment may include consideration of maintaining factors, an increase in one-on-one time that parents spend with their child, a boost of encouragement and a reduction of linguistic demands for the child [52], and emotional support for the parents.

Our results do not enable us to distinguish the potential effect of treatment from spontaneous recovery. Spontaneous recovery in the general population at 36 months post onset has been estimated to be 63% or higher [11]. An estimate of the mean time since onset of stuttering at the endpoint in our study is 33 months. Thus, our percentages of children classified as non-stuttering exceed this estimate by about 10% (p = .02; based on statistical test for comparing two proportions from different populations). Furthermore, the chance of spontaneous recovery in our clinical study population is likely to be lower than in the general population, because this chance is known to diminish after 12 to 18 months [11,14] and 56% of children within our study stuttered for at least 12 months.

Strengths of our study are the large sample size with minimal loss to follow-up, the broad range of outcome measures, the large number of measurement moments, and the relatively long follow-up period (double the time in Jones et al. [33]). Participating therapists in the RESTART-study worked in usual-care centers throughout the Netherlands. Thus, the treatments were studied in a variety of regular clinical settings with therapists unconnected to the developers of the therapies [50,53], therefore employing a practical study design ensuring a high external validity. A limitation of our study is that a high number of children appeared ineligible for participation. Results may therefore not be fully generalizable to all preschool children presenting to a clinic with stuttering. Another limitation is that the applied follow-up time is insufficient to decide conclusively whether a child has recovered from stuttering. This requires a period of about 5 years [11,54], to account for the possibility of a relapse. Therefore, we intend to follow-up all children under study.

## Conclusions

At 18 month post treatment onset, the evidence suggests that both direct and indirect treatment for stuttering can be recommended. However, direct treatment decreased stuttering more quickly during the first three months. Future research investigating the role of client and clinician factors, the effectiveness of a combined direct and indirect approach, and the cost-effectiveness of a limitation of treatment time or frequency may shed further light on the effectiveness of stuttering treatment in preschool children.

## Supporting Information

S1 CONSORT Checklist CONSORT 2010 Checklist of Information to Include When Reporting a Randomized Trial. (PDF)

S1 Protocol Study protocol approved by the Medical Ethical Committee. (PDF)

## References

[1] Månsson H. Childhood stuttering: Incidence and development. J Fluency Disord 2000 0;25(1):47–57.

[2] Reilly S, Onslow M, Packman A, Cini E, Conway L, Ukoumunne OC, et al Natural history of stuttering to 4 years of age: a prospective community-based study. Pediatrics 2013 9;132(3):460–467. 10.1542/peds.2012-3067 23979093

[3] Chang SE, Zhu DC. Neural network connectivity differences in children who stutter. Brain 2013 10 16. 10.1093/brain/awt275 PMC3859219 24131593

[4] Sommer M, Koch MA, Paulus W, Weiller C, Buchel C. Disconnection of speech-relevant brain areas in persistent developmental stuttering. Lancet 2002 8 3;360(9330):380–383. 12241779 10.1016/S0140-6736(02)09610-1

[5] Chang SE, Erickson KI, Ambrose NG, Hasegawa-Johnson MA, Ludlow CL. Brain anatomy differences in childhood stuttering. Neuroimage 2008 2 1;39(3):1333–1344. 18023366 10.1016/j.neuroimage.2007.09.067 PMC2731627

[6] Cykowski MD, Fox PT, Ingham RJ, Ingham JC, Robin DA. A study of the reproducibility and etiology of diffusion anisotropy differences in developmental stuttering: a potential role for impaired myelination. Neuroimage 2010 10 1;52(4):1495–1504. 10.1016/j.neuroimage.2010.05.011 20471482 PMC4135434

[7] Kang C, Riazuddin S, Mundorff J, Krasnewich D, Friedman P, Mullikin JC, et al Mutations in the lysosomal enzyme-targeting pathway and persistent stuttering. N Engl J Med 2010 2 25;362(8):677–685. 10.1056/NEJMoa0902630 20147709 PMC2936507

[8] Rautakoski P, Hannus T, Simberg S, Sandnabba NK, Santtila P. Genetic and environmental effects on stuttering: a twin study from Finland. J Fluency Disord 2012 9;37(3):202–210. 10.1016/j.jfludis.2011.12.003 22682321

[9] Yairi E, Ambrose N. Epidemiology of stuttering: 21st century advances. J Fluency Disord 2013 6;38(2):66–87. 10.1016/j.jfludis.2012.11.002 23773662 PMC3687212

[10] Felsenfeld S, Kirk KM, Zhu G, Statham DJ, Neale MC, Martin NG. A study of the genetic and environmental etiology of stuttering in a selected twin sample. Behav Genet 2000 9;30(5):359–366. 11235981 10.1023/a:1002765620208

[11] Yairi E, Ambrose NG. Early Childhood Stuttering: For Clinicians by Clinicians (Austin, TX: Pro-Ed). 2005.

[12] Yairi E, Ambrose NG, Paden EP, Throneburg RN. Predictive factors of persistence and recovery: pathways of childhood stuttering. J Commun Disord 1996 Jan-Feb;29(1):51–77. 8722529 10.1016/0021-9924(95)00051-8

[13] Guitar B, Conture EG. The child who stutters: to the pediatrician 5th edition Publication no. 0023 [Internet]. 2013 [cited 2014 November 16]. Memphis, TN: Stuttering Foundation of America Available: http://www.stutteringhelp.org

[14] Ingham RJ, Cordes AK. Treatment decisions for young children who stutter: Further concerns and complexities. Am J Speech Lang Pathol 1998;7(3):10.

[15] Craig A, Blumgart E, Tran Y. The impact of stuttering on the quality of life in adults who stutter. J Fluency Disord 2009 6;34(2):61–71. 10.1016/j.jfludis.2009.05.002 19686883

[16] Koedoot C, Bouwmans C, Franken MC, Stolk E. Quality of life in adults who stutter. J Commun Disord 2011 3 27. 10.1016/j.jcomdis.2011.02.002 21536306

[17] Menzies RG, Onslow M, Packman A, O'Brian S. Cognitive behavior therapy for adults who stutter: a tutorial for speech-language pathologists. J Fluency Disord 2009 9;34(3):187–200. 10.1016/j.jfludis.2009.09.002 19948272

[18] O'Brian S, Onslow M. Clinical management of stuttering in children and adults. BMJ 2011 6 24;342:d3742 10.1136/bmj.d3742 21705407

[19] Nye C, Vanryckeghem M, Schwartz JB, Herder C, Turner HM 3rd, Howard C. Behavioral stuttering interventions for children and adolescents: a systematic review and meta-analysis. J Speech Lang Hear Res 2013 6;56(3):921–932. 10.1044/1092-4388(2012/12-0036) 23275413

[20] Starkweather CW. The epigenesis of stuttering. J Fluency Disord 2002 Winter;27(4):269–87. 12506446 10.1016/s0094-730x(02)00144-4

[21] Starkweather CW, Gottwald SR. The demands and capacities model II: Clinical applications. J Fluency Disord 1990;15(3):143–157.

[22] Onslow M, Packman A, Harrison E. The Lidcombe Program of Early Stuttering Intervention: A Clinicians's Guide. Austin, Texas: Pro-ed; 2003.

[23] Onslow M, Menzies RG, Packman A. An operant intervention for early stuttering. The development of the Lidcombe program. Behav Modif 2001 1;25(1):116–139. 11151481 10.1177/0145445501251007

[24] Jones M, Onslow M, Packman A, O'Brian S, Hearne A, Williams S, et al Extended follow-up of a randomized controlled trial of the Lidcombe Program of Early Stuttering Intervention. Int J Lang Commun Disord 2008 Nov-Dec;43(6):649–661. 10.1080/13682820801895599 18608610

[25] Gore K. The Australian budget Lidcombe proposal debate: a primer in memes [Internet]. 2015 [cited 2015, June 19]. Available: http://www.speechirl.com/blog/the-australian-budget-lidcombe-proposal-debate-a-primer-in-memes

[26] Franken MC, Putker-de Bruijn D. RESTART-DCM Method. Treatment protocol developed within the scope of the ZonMW project Cost-effectiveness of the Demands and Capacities Model based treatment compared to the Lidcombe programme of early stuttering intervention: Randomised trial [Internet]. 2014 [cited 2015, January 10]. Available: http://www.nedverstottertherapie.nl/

[27] Evans S, Royston P, Day S. Minim: allocation by minimisation in clinical trials [Internet]. [cited 2013, February 8]. Available: http://www-users.york.ac.uk/~mb55/guide/minim.htm

[28] O'Callaghan CA. OxMaR: open source free software for online minimization and randomization for clinical trials. PLoS One 2014 10 29;9(10):e110761 10.1371/journal.pone.0110761 25353169 PMC4213009

[29] Riley GD. Stuttering severity instrument for children and adults Austin: Pro-Ed; 1994. 10.1044/jshd.3703.314 5057250

[30] Clark CE, Conture EG, Walden TA, Lambert WE. Speech sound articulation abilities of preschool-age children who stutter. J Fluency Disord 2013 12;38(4):325–341. 10.1016/j.jfludis.2013.09.004 24331241 PMC3868004

[31] Yaruss JS. Converting between word and syllable counts in children's conversational speech samples. J Fluency Disord 2000 0;25(4):305–316.

[32] Ingham R. J. & Riley G. Guidelines for Documentation of Treatment Efficacy for Young Children who stutter. JSLHR 1998;41:753–770. 9712124 10.1044/jslhr.4104.753

[33] Jones M, Onslow M, Packman A, Williams S, Ormond T, Schwarz I, et al Randomised controlled trial of the Lidcombe programme of early stuttering intervention. BMJ (Clinical research ed) 2005 9 24;331(7518):659. 10.1136/bmj.38520.451840.E0 PMC1226241 16096286

[34] Sawyer J, Yairi E. The effect of sample size on the assessment of stuttering severity. Am J Speech Lang Pathol 2006 2;15(1):36–44. 16533091 10.1044/1058-0360(2006/005)

[35] EuroQol Group. EQ-5D User Guide. Basic information on how to use EQ-5D. 2009; Available: http://www.euroqol.org

[36] Vanryckeghem M, Brutten GJ, Hernandez LM. A comparative investigation of the speech-associated attitude of preschool and kindergarten children who do and do not stutter. J Fluency Disord 2005;30(4):307–318. 16246410 10.1016/j.jfludis.2005.09.003

[37] Achenbach TM, Rescorla LA. Manual for the ASEBA Preschool Forms & Profiles. Burlington, VT: University of Vermont, Research Center for Children, Youth, & Families; 2000.

[38] Boberg E, Kully D. Long-term results of an intensive treatment program for adults and adolescents who stutter. JSLHR 1994 10;37(5):1050–9. 10.1044/jshr.3705.1050 7823551

[39] Newcombe RG. Two-sided confidence intervals for the single proportion: comparison of seven methods. Stat Med 1998 4 30;17(8):857–872. 9595616 10.1002/(sici)1097-0258(19980430)17:8<857::aid-sim777>3.0.co;2-e

[40] Wilson EB. Probable inference, the law of succession, and statistical inference. J. Am Statist Assoc 1927;22(158):209–212.

[41] Hayes AF, Krippendorf K. Answering the call for a standard reliability measure for coding data. Commun Methods Meas 2007;1(1):77–89.

[42] Hayes AF. Spss-macro for calculating Krippendorff’s alpha [Internet]. 2013 [cited 2014, September 20]. Available: http://www.afhayes.com

[43] Holm S. A simple sequentially rejective multiple test procedure. Scand J Stat 1979:65–70.

[44] Perneger TV. What's wrong with Bonferroni adjustments. BMJ 1998 4 18;316(7139):1236–1238. 9553006 10.1136/bmj.316.7139.1236 PMC1112991

[45] Sander EK. Reliability of the Iowa Speech Disfluency Test. J Speech Hear Disord 1961 6;(Suppl 7):21–30. 13746132

[46] Altman DG. Some common problems in medical research. Practical statistics for medical research 1991;1:396–403.

[47] Imel Z, Wampold BE. The Importance of Treatment and the Science of Common Factors in Psychotherapy In: Brown SD, Lent RW, editors. Handbook of counseling Psychology. 4th ed. New York: John Wiley & Sons Inc.; 2008 p. 249.

[48] Luborsky L, Singer B, Luborsky L. Comparative studies of psychotherapies. Is it true that "everywon has one and all must have prizes"? Arch Gen Psychiatry 1975 8;32(8):995–1008. 239666 10.1001/archpsyc.1975.01760260059004

[49] Bernstein Ratner N. Evidence-based practice in stuttering: Some questions to consider. J Fluency Disord 2005;30(3):163–188. 15961152 10.1016/j.jfludis.2005.04.002

[50] Hayhow R. Does it work? Why does it work? Reconciling difficult questions. Int J Lang Commun Disord 2011 3;46(2):155–168. 10.3109/13682822.2010.490572 21401814

[51] Donaghy M, Harrison E, O'Brian S, Menzies R, Onslow M, Packman A, et al An investigation of the role of parental request for self-correction of stuttering in the Lidcombe Program. Int J Speech Lang Pathol 2015 3 12:1–7. 10.3109/17549507.2015.1016110 25763524

[52] Bernstein Ratner N, Guitar B. Treatment of Very Early Stuttering and Parent-Administered Therapy: the State of the Art In: Bernstein Ratner N, Tetnowski JA, editors. Current Issues in Stuttering Research and Practice. 1st ed.: Psychology Press; 2006.

[53] Robey RR. A five-phase model for clinical-outcome research. J Commun Disord 2004 Sep-Oct;37(5):401–411. 15231420 10.1016/j.jcomdis.2004.04.003

[54] Conture EG. Treatment efficacy: stuttering. J Speech Hear Res 1996 10;39(5):S18–26. 8898263 10.1044/jshr.3905.s18

