# Supplementary material for: Direct versus Indirect Treatment for Preschool Children who Stutter: The RESTART Randomized Trial
Source: PLoS One. 2015 Jul 28;10(7):e0133758. doi: 10.1371/journal.pone.0133758 (PMC4517884; doi:10.1371/journal.pone.0133758)
Supplement: S1 Protocol — (PDF) [file pone.0133758.s002.pdf]

De Centrale Commissie Mensgebonden Onderzoek

Sluiten | Printen | Acties | Info

ABR-formulier

Formulier voor medisch-ethische beoordeling en registratie

ABR-formulier, versie februari 2006

Onderzoeksdossiernummer

ABR Nummer	**16180**	Status	**Definitief**
Versie	**01**	Status per	**29-01-2007**
Jaar	**07**		
Dossiernummer	**NL16180.078.07**		

Het is raadzaam de ingevoerde gegevens tijdens het invullen van dit formulier tussentijds regelmatig op te slaan. Dit voorkomt eventueel verlies van gegevens bij een verbreking van de internetverbinding.

Klik een link om naar een sectie te springen:

A. Openbaar maken gegevens medisch wetenschappelijk onderzoek
B. Administratief
C. Onderzoek
D. Proefpersonen
E. Voor- en nadelen
F. Informatie en privacy
G. Financieel
H. Publicatiebeleid
I. Indiening en beoordeling
J. Aanvullende opmerkingen
K. Samenvatting
L. Ondertekening
Wijzigingsgeschiedenis

A. Sectie - Openbaar maken gegevens medisch wetenschappelijk onderzoek

A1. De CCMO streeft naar meer transparantie over (de beoordeling van) medisch-wetenschappelijk onderzoek met mensen, geslachtscellen, embryo's en foetussen in utero. De verrichter (opdrachtgever/sponsor) kan hier aangeven toestemming te verlenen voor de openbaarmaking van de antwoorden op de vragen gemarkeerd met een wereldbol en de samenvatting bij dit formulier via de CCMO-website, dan wel de openbaarmaking te weigeren:

de verrichter verleent de CCMO toestemming voor de bovengenoemde openbaarmaking

Ga naar boven

B. Sectie - Administratief

B1. Betreft het onderzoek met geneesmiddelen (inclusief gentherapie, somatische celtherapie, GGO's, zie verder toelichting) als bedoeld in de Wet medisch-wetenschappelijk onderzoek met mensen (WMO)?

nee

B2. Houdt het onderzoek verband met een eerder door een erkende METC of door de CCMO beoordeelde studie of is het onderzoek reeds eerder bij een erkende METC ter beoordeling voorgelegd?

nee

B3. Is het protocol reeds door een andere instantie op zijn wetenschappelijke aspecten beoordeeld?

ja (stuur kopie van beoordeling mee)

B4. Is het protocol (nog) in een ander openbaar trial register geregistreerd?

nee

B5. Naam indiener/contactpersoon voor de oordelende toetsingscommissie

Achternaam indiener/contactpersoon	Franken
Titel en voorletters	Dr. M.C.
Tussenvoegsel	
Type Organisatie/Bedrijf	Universitair Medisch Centrum
Organisatie/Bedrijf	Erasmus MC, Universitair Medisch Centrum Rotterdam
Afdeling	KNO - Gehoor en Spraak Centrum
Intern adres	Sophia - Sp 1455
Adres	Dr. Molewaterplein 60
Postcode en plaats	3015 GJ Rotterdam
Land	Nederland
Telefoon	010-463 6073
Fax	
E-mail	m.franken@erasmusmc.nl

B6. Is de indiener werkzaam bij de opdrachtgever/sponsor (verrichter) van het onderzoek?

Ja

Ga naar boven

C. Sectie - Onderzoek

C1. Volledige titel van het onderzoek

C1a. In het Engels

Cost-effectiveness of the Demands and Capacities Model based treatment compared to the Lidcombe programme of early stuttering intervention: Randomised trial

C1b. In het Nederlands

Vergelijking van de kosten-effectiviteit van de op het Verwachtingen en Mogelijkheden Model gebaseerde behandeling voor stotteren met de behandeling volgens het Lidcombe Programma: een gerandomiseerde klinische trial.

C2. Verkorte titel van het onderzoek/acroniem

C2a. In het Engels

Cost-effectiveness of DCM based treatment and LP for early stuttering

C2b. In het Nederlands

Vergelijking kosten-effectiviteit van DCM- en LP-behandeling

C3. Trefwoorden (maximaal 4, plaats elk trefwoord op een aparte regel)

C3a. In het Engels

Cost-effectiveness
Randomised trial
Stuttering
Treatment

C3b. In het Nederlands

Kosten-effectiviteit
Gerandomiseerde trial
Stotteren
Therapie

C4. Beschrijf het belang van het onderzoek en de beoogde toepassing van de resultaten (verwijs eventueel naar de relevante pagina's in het protocol).

Zie ZonMW aanvraag Relevantie/Relevance, p. 6

C5. Wat is de hoofdvraagstelling van het onderzoek?

Is the Lidcombe programme for early stuttering intervention more cost-effective than the Demands and Capacities Model based treatment ?

C6. Betreft het onderzoek een multicenter-onderzoek?

ja - alleen in Nederland

C7. Is er bij multicenter-onderzoek sprake van een coördinerend onderzoeker?

ja namelijk — Naam onderzoeker:
Dr. MC Franken en Dr. EA Stolk

C8. Wie is/zijn medisch verantwoordelijk voor de proefpersonen die deelnemen aan het onderzoek

De huisarts van de proefpersonen, als verwijzer, en de behandelend logopedist.

C9. In welk centrum/welke centra (incl. huisartsenpraktijken) in Nederland wordt het onderzoek uitgevoerd? Geef per centrum op: aantal proefpersonen, naam hoofdonderzoeker, naam onafhankelijk arts (u dient het formulier eerst op te slaan).

Centrum Informatie

Centrum	Proefpersonen	Hoofdonderzoeker	Onafhankelijk arts
Centrum voor Logopedie en Stottertherapie	12	[illegible]	
Centrum voor Stottertherapie	12		
Centrum voor Stottertherapie	31		
Logopedie Almere-haven	9		
Logopediepraktijk M.P. de Boer-Groen	18		
Logopediepraktijk Naarden-Bussum	7		
Logopediepraktijk "de Streek"	3		
Logopedisch Centrum Blerick	15		
Maatschap voor Logopedie & Stottertherapie	12		
Praktijk voor Logopedie	12		
Praktijk voor Logopedie en Stotteren	12		
Stottercentrum Rijnland	12		
Stottercentrum Twente	25		
Stottercentrum Zutphen	7		
Stottercentrum Zwolle	37		

C10. Betreft het onderzoek met:
mensen

C11. Beoogd totaal aantal proefpersonen/(rest)embryo's/foetussen in utero:
C11a. In Nederland
196

C12. Op welke pagina van het onderzoeksprotocol wordt een onderbouwing gegeven van het aantal proefpersonen/(rest-)embryo's/foetussen in utero?
pagina's 10-11

C13. Onderzoeksgebied
therapie

C14. Type onderzoek
interventie-onderzoek

C15. In welke fase kan het onderzoek worden ingedeeld?
niet van toepassing

C19. Is er sprake van (nog) een andere interventie dan met geneesmiddelen zoals bedoeld in de Wet medisch-wetenschappelijk onderzoek met mensen (WMO) (zie toelichting)?
Ja

C19a.

C19b.

C19[illegible]. [illegible]ventie, namelijk treatment for early stuttering

In het Nederlands: stottertherapie voor jonge kinderen

C20. Is/zijn er (een) controlegroep (en)?
nee

C21. Betreft het een gerandomiseerd onderzoek?
ja

namelijk open, parallel

C22. Op welke klasse(n) van aandoeningen heeft het onderzoek betrekking (maximaal 3)

overig, namelijk
spraakstoornis

C23. Geef twee synoniemen voor de aandoening die bestudeerd wordt, waarvan tenminste één lekenterm.

In het Engels: stuttering
In het Nederlands: stotteren

C24. Beoogde start- en einddatum van het onderzoek
C24a. Start Datum (dd-mm-jjjj) 01-05-2007
C24b. Eind Datum (dd-mm-jjjj) 01-05-2010

Ga naar boven

D. Sectie - Proefpersonen

D1. Is er een proefpersonenverzekering conform de WMO-eisen afgesloten of wordt aan de oordelende toetsingscommissie ontheffing gevraagd?
ontheffing van de verzekering wordt gevraagd

D2. Gezonde proefpersonen en/of patiënten

Patiënten
Aantal 196

D3. Welke specifieke populaties worden voor deelname aan het onderzoek benaderd?
Kinderen t/m zes jaar die stotteren

D4. Voornaamste inclusiecriteria

D4a. In het Engels
age at recruitment 3-6 years, frequency of stuttering at least 3.3% Stuttering Like Disfluencies , type of Stuttering Like Disfluencies cf. Yairi & Ambrose (2005), and proficiency in Dutch for children and parents
D4b. In het Nederlands
het kind is tussen 3 en 6 jaar oud, stottert op ten minste 3.3% van de lettergrepen (cf. Yairi & Ambrose, 2005) en het kind en de ouders spreken vloeiend Nederlands.

D5. Voornaamste exclusiecriteria

D5a. In het Engels
onset of stuttering in the six months before recruitment, treatment for stuttering during the previous 12 months, and diagnosed neurological, learning, emotional or behavioral disorder.
D5b. In het Nederlands
het stotteren is begonnen binnen 6 maanden voor aanmelding bij de logopedist, de kinderen hebben de afgelopen 12 maanden al therapie voor het stotteren gehad, er is een neurologisch, leer-, emotioneel of gedragsprobleem vastgesteld.

D6. Bij welke categorie proefpersonen wordt het onderzoek uitgevoerd (meerdere antwoorden mogelijk)
jonger dan 12 jaar **(ga naar vraag D8)**

D8. Waarom wordt het onderzoek niet met meerderjarige/wilsbekwame proefpersonen uitgevoerd?
Het betreft een behandeling voor kinderen in de voorschoolse leeftijd (< groep 3) en kan dus alleen bij deze categorie worden uitgevoerd.

D9. Hoe is gewaarborgd dat feitelijk verzet van de proefpersoon gesignaleerd wordt en dat er rekening mee wordt gehouden (verwijs eventueel naar de relevante pagina's in het protocol en/of de schriftelijke informatie voor de patiënt/wettelijke vertegenwoordiger)?

zie patienteninformatie
pagina 3 : Wat gebeurt er als u niet wilt meedoen?
pagina 4: Vrijwillige deelname

D10. Verkeren (sommige) proefpersonen in een afhankelijkheidssituatie ten opzichte van de onderzoeker of degene die de deelnemers werft? (lees de toelichting voor voorbeelden wanneer er sprake kan zijn van een afhankelijkheidssituatie)

ja

D10a. Zo ja, waardoor?

De logopedist waartoe de ouder zich went zal vragen om medewerking aan het onderzoek. Er is echter geen sprake van een 'eigen logopedist' zoals een 'eigen huisarts': de ouder heeft alle vrijheid om zich tot een andere logopedist te wenden.

D10b. Waarom wordt het onderzoek juist met deze proefpersonen uitgevoerd en hoe worden de belangen van de proefpersonen gewaarborgd?

Het onderzoek kan alleen worden gedaan met jonge kinderen die stotteren en waarvoor de ouders logopedische behandeling willen; zij zijn alleen op de beschreven manier te includeren. Voordeel voor de deelnemende ouders zelf kan zijn dat de resultaten van de behandeling uitgebreid worden geevalueerd. De belangen van de proefpersonen worden gewaarborgd doordat er sprake is van vrijwillige deelname en geen verandering van zorg als de ouder niet mee wil doen.

D11. Waaruit bestaat de vergoeding voor de proefpersonen?

geen vergoeding

D12. Is deze vergoeding afhankelijk van bepaalde voorwaarden, bijvoorbeeld het voltooien van (een deel van) het onderzoek?

niet van toepassing

Ga naar boven

E. Sectie - Voor- en nadelen

E1. Wordt er bij dit onderzoek een rechtstreeks therapeutisch effect beoogd bij de proefpersonen / patiënten?

ja (therapeutisch onderzoek)

E1a. Zo ja, geef kort aan waaruit dit therapeutisch effect bestaat.

Herstel van (normaal-)vloeiend spreken

E2. Waaruit bestaat de belasting van het onderzoek (en een eventueel daaraan voorafgaande keuring) voor de proefpersonen?

Tijdbeslag	per bezoek	nvt
	totaal	maximaal 8,5 uur
	totale duur van de studie voor de individuele proefpersoon	18 maanden

E3. Worden de proefpersonen in verband met het onderzoek in het ziekenhuis opgenomen of wordt een opname verlengd?

nee

E4. Beschrijf in hoeverre proefpersonen worden onderworpen aan handelingen dan wel een gedragswijze krijgen opgelegd, zoals vragenlijst, interviews, lichamelijk/psychologisch onderzoek, ontzegging, dieet (voor invasieve ingrepen: zie vraag E6)

Aan de ouders van de proefpersonen wordt gevraagd om thuis audio-opnames van gesprekken met hun kind te maken en hun wordt gevraagd om vragenlijsten in te vullen.

E5. Worden de proefpersonen getest op bepaalde aandoeningen/condities?

ja (motiveer)

namelijk taalstoornis

E6. Welke extra (invasieve) ingrepen (anders dan bij de standaard behandeling) moeten de proefpersonen in het kader van het onderzoek ondergaan:

Niet van toepassing

E7. Welke maatregelen zijn genomen om (eventuele) risico's van de bijkomende ingrepen te vermijden en nadelige gevolgen te beperken?

NVT

E8. Hoe is de spreiding van de ingrepen in de tijd, met name afname van bloed en ander lichaamsmateriaal (verwijs eventueel naar de flow chart in het protocol)?
NVT

E9. Geef aan welke risico's er voor proefpersonen zijn verbonden aan deelname aan het onderzoek.
Geen risico

E9a. Geef op grond van uw eigen afweging aan waarom het uitvoeren van het onderzoek, in het licht van de belasting en/of risico's die voor proefpersonen aan deelname verbonden zijn, gerechtvaardigd is?
Belasting van maximaal 8,5 uur tijdsinvestering, die tevens een goede evaluatie voor de betrokkenene zelf tegenover een belangrijke uitbreiding van de kennis in therapiekeuze die beredeneerd klinisch handelen mogelijk maakt vind ik gerechtvaardigd, zeker ook omdat de ouders kunnen weigeren om de extra tijdsinvestering te geven.

E10. Indien het onderzoek bij minderjarige en/of wilsonbekwame proefpersonen wordt uitgevoerd en geen direct therapeutisch effect wordt beoogd: waarom kunnen belasting en risico's als minimaal worden beschouwd? (Verwijs eventueel naar de relevante pagina's in het protocol.)
niet van toepassing

E11. Kan de eventuele therapie na beëindiging van het onderzoek worden voortgezet?
ja (motiveer)

in welk kader en voor hoelang?	therapie kan normaal worden voortgezet bij de behandelend logopedist

E12. Heeft deelname aan het onderzoek voor de proefpersoon tot gevolg dat van de standaardbehandeling of -diagnostiek kan worden afgeweken of deze kan worden uitgesteld?
ja

E12a. Zo ja, waaruit bestaat de afwijking of het uitstel en waarom is afwijking/uitstel
Er wordt in het onderzoek gerandomiseerd over de standaardbehandeling en een nieuwe behandeling. De nieuwe behandeling lijkt effectiever maar is wel duurder omdat er meer behandelingen mee gepaard gaan, gemiddeld genomen.

Ga naar boven

F. Sectie - Informatie en privacy

F1. Hoe worden de proefpersonen geworven en door wie (onderzoeker, behandelend arts, andere persoon) wordt de proefpersoon/wettelijke vertegenwoordiger geïnformeerd en om toestemming gevraagd?
De proefpersonen worden door de behandelend logopedist uitgenodigd om mee te doen aan het onderzoek als ze op grond van de inclusiecriteria geschikt zijn om mee te doen. Ook worden ze ge"informeerd door de behandelend logopedist (op basis van de schriftelijke patientinformatie die de onderzoekers aanleveren) en wordt hun door de behandelend logopedist toestemming gevraagd.

F2. Hoeveel bedenktijd krijgen de proefpersonen/wettelijke vertegenwoordigers om te beslissen over deelname?
Zolang ze wensen.

F3. Wordt de huisarts, behandelend specialist en/of apotheker van de proefpersoon geïnformeerd over diens deelname aan het onderzoek?
ja (de proefpersoon dient hiervoor toestemming te geven)

F4. Worden persoonsgegevens gecodeerd?
ja

F4a. Zo ja, hoe is deze codering opgebouwd?
Ja, volgnummer in onderzoek

F4b. Wie heeft toegang tot de sleutel van deze code?
De onderzoekers

F4c. Wie hebben toegang tot de brondocumenten en eventuele andere tot de persoon herleidbare gegevens?
De onderzoekers

F5. Hoe wordt het lichaamsmateriaal gedurende het onderzoek bewaard?
niet van toepassing

F6. Wordt afgenomen lichaamsmateriaal na afloop van het onderzoek vernietigd?

niet van toepassing

F7. Kunnen proefpersonen na afloop van het onderzoek opnieuw benaderd worden (bijvoorbeeld voor nader onderzoek of follow-up)?

ja

F7a. Wordt aan de proefpersoon hiervoor in het voorliggende onderzoek toestemming gevraagd?

ja

Ga naar boven

G. Sectie - Financieel

G1. Door welke geldstroom wordt het onderzoek gefinancierd?

tweede geldstroom (NWO of KNAW), namelijk:

ZonMW

G2. Wordt het onderzoek (mede) gefinancierd door de industrie/bedrijven?

nee

G2a. Geef de namen van industrie/bedrijven indien anders dan de opdrachtgever/verrichter (max 5).

Industrie/Bedrijf 1

G3. Ontvangt de arts/onderzoeker persoonlijk (afgezien van zijn/haar normale salaris) een vergoeding voor de uitvoering van het onderhavige onderzoek?

ja

G3a. Is hiervoor een contract met de sponsor/subsidiegever afgesloten?

ja

Zo ja, tussen welke partijen en personen is het contract afgesloten (evt contract bijsluiten)?
In de begroting van de subsidieaanvraag is de volgende vergoeding aan de deelnemende behandelende logopedisten opgenomen: (1) Vergoeding van 130 Euro per patient waarvan alle metingen zijn gecompleteerd als tegemoetkoming in de tijdsinvestering die meedoen aan dit onderzoek vergt; (2) Vergoeding van Euro 170 Euro per gevolgde intervisiebijeenkomst in Utrecht (tegemoetkoming in de resikosten en vacatiegeld). Verder wordt aan de behandelend logopedisten video- en audioapparatuur ter beschikking gesteld om de benodigde metingen voor het onderzoke te kunnen doen.

G3b. Wat is de hoogte van de vergoeding die de arts/onderzoeker **persoonlijk** ontvangt voor de uitvoering van het van het onderzoek?

Per patiënt of proefpersoon (bedragen in euro's)

€ 130,00

G4. Heeft/hebben de onderzoeker(s) gedurende de afgelopen vijf jaar op een of andere wijze een persoonlijke financiële relatie (gehad) met de verrichter/sponsor van het huidige onderzoek?

nee

Ga naar boven

H. Sectie - Publicatiebeleid

H1. Zijn door de opdrachtgever/subsidiegever bepaalde voorwaarden gesteld aan het openbaar maken van de onderzoeksresultaten?

nee

Ga naar boven

I. Sectie - Indiening en beoordeling

I1. Sla het formulier eerst op en selecteer vervolgens de toetsingscommissie die het oordeel geeft in de zin van de WMO

Commissie

METC Erasmus MC, Universitair Medisch Centrum Rotterdam (Rotterdam)

Ga naar boven

J. Sectie - Aanvullende opmerkingen

Aanvullende opmerkingen

Ga naar boven

K. Sectie - Samenvatting

Bij dit formulier dient een Nederlandstalige samenvatting van het protocol gevoegd te worden. Indien toestemming is verleend voor de openbaarmaking dient tevens een Engelstalige samenvatting te worden bijgevoegd.

De samenvatting is een onderdeel van het formulier en dient een duidelijke beschrijving te geven van de centrale vraagstelling die men wil beantwoorden met het onderzoek. In de samenvatting wordt in het kort de onderzoeksopzet, de studie populatie, de methoden die gebruikt worden in het onderzoek. Ten slotte dient de belasting en het risico voor de proefpersoon beschreven te worden. De samenvatting is maximaal 1 A4 lang.

K1. Nederlandse Samenvatting

Titel onderzoek
Vergelijking van de kosten-effectiviteit van de op het Verwachtingen en Mogelijkheden Model gebaseerde behandeling voor stotteren met de behandeling volgens het Lidcombe Programma: een gerandomiseerde klinische trial.

Achtergrond van het onderzoek:

Momenteel krijgen alle jonge kinderen (kinderen jonger dan zes jaar oud) die stotteren in Nederland logopedie, in tegenstelling tot ongeveer 20 jaar geleden. Toen werd eerst afgewacht tot kinderen zes jaar oud waren. In Nederland is behandeling op basis van het Demands and Capacities Model de standaard behandeling. In 2000 is de Australische standaardmethode in Nederland ingevoerd. Deze methode lijkt effectiever, maar is wel duurder. In deze studie wordt de kosten-effectiviteit van beide methodes vergeleken.

Doel van het onderzoek:

Het doel van deze studie is om de kosten-effectiviteit van twee therapieën voor stotteren bij in jonge kinderen te vergelijken. Het gaat om een behandeling die is gebaseerd op het zogenaamde 'Demands and Capacities Model' (de huidige standaard in Nederland) en het zogenaamde 'Lidcombe programma'

Onderzoeksopzet:

De studie wordt uitgevoerd in een klinische trial met een naturalistisch design. Toewijzing aan de behandelarmen gebeurt random; de uitkomst analyse is geblindeerd. Metingen worden verricht op baseline, en 3, 6, 12 en 18 maanden na aanvang van de behandeling.

Onderzoekspopulatie:

Kinderen tussen 3;0 en 6;11 jaar oud die stotteren en zich voor behandeling aanmelden bij de logopedist.

Interventie (indien van toepassing):

1. Behandeling gebaseerd op ht Demands & Capacities Model
2. Lidcombe Programma

Primaire onderzoeksvariabelen/uitkomstmaten:

- Percentage kinderen dat 18 maanden na aanvang van de therpaie is hersteld van het stotteren
- Stotterfrequentie
- Child Health Questionnaire
- Kosten per kind dat geneest.

Secundaire onderzoeksvariabelen/uitkomstmaten (indien van toepassing):

- Health Utilities Index

- Child behaviour Checklist, CBCL

Omschrijving en inschatting van belasting en risico (indien van toepassing):

Maximaal 8,5 uur tijdsinvestering voor invullen vragenlijsten en maken audio-opnames

K2.Engelse Samenvatting

Title of the study
Cost-effectiveness of the Demands and Capacities Model based treatment compared to the Lidcombe programme of early stuttering intervention: Randomised trial

Background of the study:

Currently, treatment is provided to all pre-school stuttering children, to prevent stuttering becoming a chronic, handicapping condition in some of them. In the Netherlands, most children are treated with the Dutch standard, the DCM. Experienced clinicians spend about 9 sessions of treatment for each child in the Demands and Capacities based treatment. Recently, the Australian standard treatment, the Lidcombe Programme has been introduced in the Netherlands. The lidcombe Programma takes mostly at least about 18 sessions, so twice as much. But, the clinicail impression is that the results with the Lidcombe Programme are significanty better. Yet, about 50 clinicians have been trained to deliver treatment along the LP approach also. The difference in empirical support for LP and DCM suggests that in the long-term, LP might be more cost-effective than the current standard, DCM: the proportion of pre-school stuttering children that not only decrease their stuttering frequency but recover from stuttering after completion of the LP might be higher than for DCM, though, for higher costs. But, in the long term, for comparable costs. This study therefore explores the cost-effectiveness of the two treatments.

Objective of the study:

To evaluate the cost-effectiveness of the Demands and Capacities Model based treatment (Dutch standard) compared to the Lidcombe programme for early stuttering intervention.

Study design:

A pragmatic, open plan, parallel group, randomised trial with blinded outcome assessment.

Study population:

Pre-school stuttering children who presented to the speech clinics for treatment.

Intervention (if applicable):

1. Treatment based on the Demands & Capacities Model
2. Lidcombe Programme of early stuttering intervention.

Primary study parameters/outcome of the study:

The percentage of recovered children at 18 months post randomisation, frequency of stuttering and severity ratings, Child Health Questionnaire, costs for a recovered child.

Secundary study parameters/outcome of the study (if applicable):

Health Utility Index, Child Behaviour Check List

Nature and extent of the burden and risks associated with participation, benefit and group relatedness (if applicable):

Maximum of 8,5 hours for filling in questionaires and making audio-recordings at home by the parents.

Ga naar boven

ONDERTEKENING

De verrichter en indiener verklaren hierbij:

a. het formulier (en samenvatting) volledig en naar waarheid te hebben ingevuld;
b. de antwoorden op de vragen uit het ABR-formulier niet in strijd zijn met het bijbehorende onderzoeksdossier en onderzoekscontract

Naar waarheid getekend, door de verrichter (=opdrachtgever) door de indiener

datum	15-2-07	datum	26-2-07.
Handtekening		Handtekening	
naam	Prof dr Baatenburg de Jong	naam	Dr. MC Franken
functie	Hoofd Afdeling KNO, Hoofd-Hals chirurgie, Gehoor en Spraak Centrum	functie	Linguist-logopedist

Ga naar boven

Wijzigingsgeschiedenis

11-01-2007 10:20:12 - Franken heeft dit ABR-formulier aangemaakt

Ga naar boven

ADDENDUM BIJ HET ABR-FORMULIER VOOR TOETSING VAN MEDISCH WETENSCHAPPELIJK ONDERZOEK DOOR DE METC ERASMUS MC

Titel onderzoek: 'Cost-effectiveness of the Demands and Capacities Model based treatment compared to the Lidcombe programme of early stuttering intervention: Randomised trial' . . . .
. . . .

Algemeen:

1. Wie zijn de hoofd- en medeonderzoekers in het Erasmus MC en op welke afdeling(en) zijn zij werkzaam?
   Dr. M.C. Franken / KNO en Dr. E.A. Stolk / IMTA . . . .
   . . . .

2. Zijn onderzoeker en behandelaar dezelfde persoon?
   ☐ Ja ☒ Nee

3. Door wie en waar vindt de statistische bewerking van de data plaats?
   Door de projectuitvoerder (nog aan te stellen) gesuperviseerd door de projectbegeleiders, in het Erasmus MC. . . . . .

1. Welke partijen zijn naast de onderzoeker(s) betrokken bij de uitvoering van het onderzoek?
   (Naam van bijvoorbeeld sponsor, clinical research organization (CRO), safety monitoring board (SMB), data center, trialbureau, stichting e.d. invullen)
   Geen partijen, slechts deelnemende logopedisten in zelfstandige, perifere praktijken... . . . .

2. . . . .

Bij (mede) door de derde geldstroom gesponsord onderzoek: NVT

1. Ontvangen de bij de uitvoering van het onderzoek betrokken partijen (onderzoekers, CRO, SMB, data center, trialbureau, stichting e.d.) een financiële vergoeding van de sponsor voor de te verrichten werkzaamheden?
   ☐ Ja ☐ Nee
   Zo ja, hoe hoog is deze vergoeding voor de verschillende partijen?
   Partij: . . . . ontvangt € . . . .

2. Hoe wordt belangenverstrengeling tussen deze partijen en de sponsor tegengegaan?
   . . . .

3. Is er een afhankelijkheidsrelatie tussen deze partijen en de sponsor?
   ☐ Ja, te weten, ... ☐ Nee

Bij onderzoek met farmaceutische producten: NVT

1. Vragen inzake de lokale uitvoerbaarheid van het onderzoek:
   a. Is er een externe leverancier van de onderzoeksmedicatie?
      ☐ Ja, zie c ☐ Nee, zie b
   b. Zijn er afspraken met de ziekenhuisapotheek over de bereiding en kwaliteitsborging van de onderzoeksmedicatie?
      ☐ Ja ☐ Nee, de commissie verzoekt u contact op te nemen met de ziekenhuisapoteker (tel. 4633202).
   c. Is een Investigational Medicinal Product Dossier (IMPD) beschikbaar?
      ☐ Ja ☐ Nee
   d. Zijn er afspraken met de ziekenhuisapotheek over logistieke aspecten, waaronder levering, opslag, etikettering, ter handstelling van onderzoeksmedicatie?

☐ Ja ☐ Nee, de commissie verzoekt u contact op te nemen met de ziekenhuisapoteker (tel. 4633202).

2. Is het 'Besluit immunologisch farmaceutische producten' van toepassing?
(voor inlichtingen hieromtrent kunt u terecht bij de ziekenhuisapotheek, tel. 4633202)
☐ Ja ☐ Nee

3. Vragen inzake de toediening van onderzoeksmedicatie aan de proefpersoon in het ziekenhuis:
   a. Worden andere personen dan de arts-onderzoeker / behandelend arts belast met de toediening van de onderzoeksmedicatie?
   ☐ Ja, zie b ☐ Nee
   b. Indien toediening van de onderzoeksmedicatie plaatsvindt door een andere persoon dan de arts-onderzoeker / behandelend arts, bijvoorbeeld verpleegkundige, is de Wet BIG van toepassing, waarbij moet vastliggen dat betrokkenen deskundig en bekwaam zijn voor de toediening. Daarnaast dient er een specifieke schriftelijke instructie voor toediening van de onderzoeksmedicatie, inclusief veiligheidsinformatie (= protocol) beschikbaar te zijn.
      I. Is de andere persoon dan de arts-onderzoeker / behandelend arts uitvoerig geïnformeerd over aard en risico's van toediening van de onderzoeksmedicatie?
      ☐ Ja ☐ Nee
      II. Vindt toediening onder direct toezicht plaats van de arts-onderzoeker / behandelend arts?
      ☐ Ja ☐ Nee
      III. Is er steeds een mogelijkheid voor tussenkomst van een deskundige arts (= supervisie)?
      ☐ Ja ☐ Nee

Bij onderzoek met medische apparatuur: NVT

1. Is de medische apparatuur voor het beoogde gebruik goedgekeurd door de afdeling Medische Technologie van de Directie Informatie?
☐ Ja ☐ Nee, de commissie verzoekt u contact op te nemen met de Consultant Medische Technologie de heer Ing. N.G.C. Laheij (tel. 4635644) of met de manager van de afdeling Medische Technologie mevrouw Ir. J.N. Nathan (tel. 4634018).

2. Is de medische apparatuur geproduceerd voor de door u beoogde toepassing?
☐ Ja ☐ Nee, de commissie verzoekt u contact op te nemen met de Consultant Medische Technologie de heer Ing. N.G.C. Laheij (tel. 4635644) of met de manager van de afdeling Medische Technologie mevrouw Ir. J.N. Nathan (tel. 4634018).

Bij onderzoek met steriele en niet-steriele medische hulpmiddelen (disposables of reusables) <u>zonder</u> CE-markering: NVT

1. Is het medisch hulpmiddel ter beoordeling voorgelegd aan de Deskundige Medische Hulpmiddelen?
☐ Ja ☐ Nee, de commissie verzoekt u contact op te nemen met de Deskundige Medische Hulpmiddelen, de heer F. Raymaekers (tel. 4632448).

Bij onderzoek met steriele en niet-steriele medische hulpmiddelen (disposables of reusables) <u>met</u> CE-markering: NVT

1. Is het medisch hulpmiddel aangeschaft volgens de 'Procedure Medische Hulpmiddelen'?
☐ Ja ☐ Nee, de commissie verzoekt u contact op te nemen met de Deskundige Medische Hulpmiddelen de heer F. Raymaekers (tel. 4632448).

2. Is het medisch hulpmiddel geproduceerd voor de door u beoogde toepassing?
☐ Ja ☐ Nee, de commissie verzoekt u contact op te nemen met de Deskundige Medische Hulpmiddelen de heer F. Raymaekers (tel. 4632448).

Bij onderzoek waarbij ten behoeve van het onderzoek beeldvormende diagnostiek, zoals conventionele radiologie, Computed Tomography (CT), Magnetic Resonance Imaging (MRI), Echografie en of radiologische interventies gewenst zijn: NVT

1. Is overleg gepleegd met de afdeling Radiologie voor het uitvoeren van deze onderzoeken?
   ☐ Ja ☐ Nee, de commissie verzoekt u contact op te nemen met de Research Office Radiologie (tel. 4632277, research.radiology@erasmusmc.nl).

Bij onderzoek met stralingsbronnen (niet alleen radiofarmaca, ingekapselde radioactieve bronnen, en deeltjesversnellers, maar ook röntgentoestellen, waarbij de beeldvorming zelf geen deel uit maakt van de onderzoeksvraagstelling): NVT

1. Is in het protocol de stralingsblootstelling beschreven en is vermeld hoe groot de stralingsbelasting is?
   ☐ Ja, pagina . . . . ☐ Nee

2. Is overleg gepleegd met de algemeen stralingsdeskundige van het Erasmus MC?
   ☐ Ja ☐ Nee, de commissie verzoekt u contact op te nemen met de heer P.J.H. Kicken (tel. 4391130) en/of hem het protocol en het patiënteninformatieformulier toe te sturen.

Bij onderzoek met bloedproducten:NVT

1. Welke waarborgen zijn door de producent getroffen om zekerheid te verkrijgen dat het eindproduct virusveilig is? . . .
   . . . .

Indien vraag F4 van het ABR-formulier met 'Nee' wordt beantwoord: NVT

1. Wordt – in het kader van de verificatie van de juistheid van (medische) gegevens – inzage in op naam gestelde documenten verleend aan derden?
   ☐ Ja ☐ Nee

2. Zo ja, aan welke derden wordt inzage verleend?
   - ☐ Monitor / industrie
   - ☐ Overheid ☐ Nationaal: Inspectie voor de Gezondheidszorg
     ☐ Internationaal: FDA
   - ☐ Universiteit
   - ☐ Ziekenhuis
   - ☐ Anderszins, n.l. . . . .

3. Zijn personalia en kwalificaties van bovengenoemde derden terug te vinden in het protocol?
   ☐ Ja, pagina . . . . ☐ Nee

4. Hebben deze derden:
   - ☐ een eigen beroepsgeheim op grond van de BIG?
   - ☐ een geheimhoudingsplicht?
   - ☐ een geheimhoudingsverklaring ondertekend?
